# Measures during the COVID-19 pandemic in public primary health care in Greece: is there still a missing link to universal health coverage?

**DOI:** 10.1186/s12875-024-02392-7

**Published:** 2024-05-17

**Authors:** Efthalia Tsampouri, Konstantina Kapetaniou, Aristea Missiou, Maria Bakola, Sara Willems, Esther Van Poel, Athina Tatsioni

**Affiliations:** 1 Research Unit for General Medicine and Primary Health Care, Faculty of Medicine, School of Health Sciences, University of Ioannina, Ioannina, Greece; 2 Quality and Safety Ghent, Department of Public Health and Primary Care, Ghent University, Ghent, Belgium

**Keywords:** COVID-19, Greece, Family medicine, PRICOV-19 study, Primary health care, Quality of care, Rural setting

## Abstract

**Background:**

The PRICOV-19 study aimed to assess the organization of primary health care (PHC) during the COVID-19 pandemic in 37 European countries and Israel; and its impact on different dimensions of quality of care. In this paper, we described measures taken by public PHC centers in Greece. Additionally, we explored potential differences between rural and non-rural settings.

**Methods:**

The study population consisted of the 287 public PHC centers in Greece. A random sample of 100 PHC centers stratified by Health Region was created. The online questionnaire consisted of 53 items, covering six sections: general information on the PHC center, patient flow, infection prevention, information processing, communication to patients, collaboration, and collegiality.

**Results:**

Seventy-eight PHC centers (78%) - 50 rural and 28 non-rural – responded to the survey. Certain measures were reported by few PHC centers. Specifically, the use of online messages about complaints that can be solved without a visit to the PHC center (21% rural; and 31% non-rural PHC centers), the use of video consultations with patients (12% rural; and 7% non-rural PHC centers), and the use of electronic medical records (EMRs) to systematically identify the list of patients with chronic conditions (5% rural; and 10% non-rural PHC centers) were scarcely reported. Very few PHC centers reported measures to support identifying and reaching out to vulnerable population, including patients that may have experienced domestic violence (8% rural; and 7% non-rural PHC centers), or financial problems (26% rural; and 7% non-rural PHC centers). Providing administrative documents to patients through postal mail (12% rural; and 21% non-rural PHC centers), or regular e-mail (11% rural; and 36% non-rural PHC centers), or through a secured server (8% rural; and 18% non-rural PHC centers) was rarely reported. Finally, providing information in multiple languages through a PHC website (12% rural PHC centers only), or an answering machine (6% rural PHC centers only), or leaflets (3% rural PHC centers only; and for leaflets specifically on COVID-19: 6% rural; and 8% non-rural PHC centers) were lacking in most PHC centers.

**Conclusion:**

Our study captured measures implemented by few PHC centers suggesting potential priority areas of future improvement.

**Supplementary Information:**

The online version contains supplementary material available at 10.1186/s12875-024-02392-7.

## Introduction

As healthcare systems navigated through the COVID-19 pandemic and prepared for the next phase, the stress put on primary health care (PHC) was more pronounced than ever. The Astana Declaration, in 2018, had already described three components of PHC as a vehicle towards health for all: integration of primary care and essential public health functions; empowerment of people and communities; multisectoral policy and action [1]. The COVID-19 outbreak reaffirmed the need for a PHC that shares these features, as indicated by examples of successful pandemic management [2–4]. Moreover, strong PHC and community care has been outlined as a prerequisite so that health care systems can build resilience to crises of this magnitude [5, 6].

For countries with an already weak PHC system, inevitably the circumstances were more challenging. In the case of Greece, when the pandemic started, a well-structured PHC system was lacking [7]. Health service delivery remained fragmented, mainly focused on disease management [8]. Previous reforms only partially addressed the goal of universal health coverage [9], and the country reported a high level of unmet needs [10], even before the outbreak [11]. Acknowledging these gaps, voices were raised early in the pandemic outbreak to highlight the urgency to strengthen Greece’s PHC and foster its role [12, 13]. In this direction, the government implemented several measures so that public PHC could be more actively involved in the country’s response to the pandemic [10, 14]. For example, certain PHC centers were exclusively assigned to identify and manage COVID-19 cases, while others served people with chronic conditions [10, 14].

To the best of our knowledge, limited nationwide evidence exists on how PHC centers operated within this new framework in Greece. Therefore, we sought to address this gap, by participating in the cross-sectional PRICOV-19 study, which aimed to assess organization of general PHC centers (PHC centers) during the pandemic in 37 European countries and Israel, as well as the pandemic’s impact on different dimensions of quality of care [15]. Our primary aim was to describe measures taken during the COVID-19 pandemic by public PHC centers in Greece. As a secondary aim, we explored potential differences in these measures between rural and non-rural public PHC centers, since previous studies suggested that awareness of the response measures to the COVID-19 pandemic and management protocol requirements that were in place might have been different between rural and urban areas [16].

## Methods

### Ethics approval

The study was conducted according to the guidelines of the Declaration of Helsinki. The Research Ethics Committee of Ghent University Hospital approved the protocol of the PRICOV-19 study (BC-07617). The data collection in Greece was approved by the University of Ioannina Ethics Committee (49,107/16-12-2020). It was also approved by the Scientific Committees of the Administrations of the seven Health Regions in Greece. All participants gave informed consent.

### Study design

PRICOV-19 is a cross-sectional multi-country study using an online questionnaire for PHC centers. This international study was set up by the expertise center “Quality and Safety Ghent” (Department of Public Health and Primary Care, University of Ghent) and in collaboration with the two WONCA networks EQuiP (European Society for Quality and Patient Safety in General Practice/Family Medicine) and EGPRN (European General Practice Research Network), and the EFPC (European Forum for Primary Care). The Research Unit for General Medicine and Primary Health Care (University of Ioannina, Faculty of Medicine) conducted the Greek study part. This article focuses on the results for PHC centers in Greece.

### Study questionnaire

The initial questionnaire was developed in Flemish for the pilot study in Flanders (Belgium). Afterwards, the final instrument was forward-backward translated to English, at Ghent University (Belgium), and sent to all participating countries, which could add up to three country-specific questions. A detailed description of the questionnaire development has been reported elsewhere [15]. The PRICOV-19 study aimed to describe how GP practices across 37 European countries and Israel were organized during the COVID-19 pandemic to guarantee safe, effective, efficient, patient-centered, and equitable care [15]. However, due to the heterogeneity in recruitment strategies among countries balancing between a consequent strategy according to the study protocol and adaption to the local situation, comparisons between countries might be difficult to be interpreted (Tatsioni A, Groenewegen P, Van Poel E, Vafeidou K, Assenova R, Hoffmann K, Schaubroeck E, Stark S, Tkachenko V, Willems S: Recruitment, data collection, participation rate, and representativeness of the international cross-sectional PRICOV-19 study across 38 countries, submitted). Thus, separate publications by countries may better describe the situation in each country and account for distinct issues that may have affected each country’s response to the pandemic.

To collect data in Greece, we proceeded with translation in Greek, back-translation in English, and cultural adaptation. Before launching the survey in Greece, we also piloted the questionnaire in five PHC employees and further adapted it according to their comments (Appendix 1). The final questionnaire consisted of 53 items divided into six sections: general information on the PHC center, patient flow, infection prevention, information processing, communication to patients, collaboration, and collegiality (Appendix 2).

### Sample selection

The study population consisted of the PHC centers in Greece. These facilities are government funded and may supervise smaller settings in the neighboring area. Public PHC centers along with their satellite solo GP practices or their satellite local health units staffed with multidisciplinary health teams consisting of family doctors, nurses, health visitors, social workers, and administrative staff are dispersed throughout the country, in rural and non-rural areas. Besides GPs, internists and other specialists may work in PHC centers. Since a GP is not mandatory for PHC centers, it is possible that certain PHC centers are staffed only by specialists and other health professionals. The sampling frame included all the 287 public PHC centers in Greece, which were identified in the website lists of the Administrations of the seven Health Regions. Updated lists were obtained after a telephone communication with each of the seven Health Region Administrations. We excluded private PHC providers since an updated list was not available.

A random sample of 100 PHC centers stratified by Health Region was created for the study. We used a random number generator on the list of 287 centers in alphabetical order. A minimum of 50 centers in the final sample was set as a target, according to the PRICOV-19 study requirements. However, if the PHC centers that refused to participate during our contact had exceeded 50%, they would have been replaced by other centers from the same Health Region to reach the target sample size. Since there is no official list that identifies rural, and non-rural public PHC centers in Greece, it was difficult to decide upfront which PHC centers satisfy the criteria for rurality. In the results, we were able to estimate the percentage of rural, and non-rural PHC centers only for the PHC centers in our sample, based on what responders reported in the survey.

### Data collection

Data collection took place between January 12th and March 23rd, 2021. A research team member (ET, KK, AM, and MB) contacted the Head of each PHC center by phone, requesting to identify one of the employees – preferably a family physician – to fill in the questionnaire. According to the PRICOV-19 study protocol, the questionnaire could be also completed by any other staff member that was familiar with the practice organization. On the same day, our team member contacted the employee by phone to explain the process and record his/her e-mail address to send the survey link. In case the suggested person refused to participate, we asked whether another employee in the same center might be available. For PHC centers with at least one person available, the survey link was sent through e-mail kindly requesting the employee to complete the questionnaire within the following 2 days and to send back a notification e-mail. If we did not receive a notification e-mail within 48 hours, we contacted the employee by phone and kindly reminded him/her of the task. A second attempt by phone that failed was considered a refusal. In addition, we recorded potential reasons for refusal employees reported during the data collection process. The Research Electronic Data Capture (REDCap) platform [17] was used to host the questionnaire in all languages, send out invitations to the national samples of PHC centers, and securely store the answers from the participants.

### Statistical analysis

Ghent University was responsible for the data cleaning. Our analyses were based on the cases that filled in at least the first part of the questionnaire and submitted the survey, and thus considered as responders. Data were presented as absolute numbers and percentages for binary and categorical variables and as median with interquartile range (IQR) for continuous variables. We presented separately items for PHC centers in rural areas (i.e., those reported to be located in rural and mixed semi-rural settings), and for centers in non-rural settings (i.e., those reported to be located in big cities, suburbs, and small towns). To facilitate interpretation, we transformed the 5-point Likert scale responses into two category responses, merging responses of the two upper points as a “positive” answer and the responses of the three lower points as a “negative” answer (Appendix 3). For the secondary objective, comparisons between rural and non-rural PHC centers were performed using the Pearson Chi-Square test or Fisher’s Exact test for discrete variables and the Mann-Whitney test for continuous variables as appropriate. For all comparisons, we considered statistically significant a *P-*value less than 0.05. All analyses were performed with IBM SPSS Statistics for Windows, Version 26.0. (IBM Corp, Armonk, NY, USA), and Microsoft Excel, MS Office 2019 (Microsoft Corp, Redmond, Washington, USA).

## Results

### Study flow

Out of the 100 PHC centers in our random sample, 3 were considered as ineligible since they were merged to neighboring hospitals and were no longer functioning as separate facilities. Out of the 97 eligible PHC centers, we could not reach one center despite multiple efforts. Eighteen centers declined our invitation; 6 centers refused to participate due to excessive workload; and 12 PHC centers did not provide any reason for their refusal to participate. The remaining 78 PHC centers that responded to the survey included 50 rural and 28 non-rural centers.

### Characteristics of the PHC centers that filled in the survey

Generally, there were no statistically significant differences between rural and non-rural PHC centers regarding PHC center and population characteristics (Table 1; Table 2). However, rural PHC center were more likely to have a receptionist as compared to non-rural PHC centers [40 (80%) rural; 14 (50%) non-rural; *P*-value 0.01]. Rural and non-rural centers reported a median number of 25 (IQR 6, 40) and 23 (IQR 3, 61) employees per PHC center, respectively; and a median number of 6 (IQR 3,9) GPs per rural PHC center and 6 (IQR 1,16) GPs per non-rural PHC center. Most PHC centers counted practice managers [40 (80%) rural; 18 (64%) non-rural], GPs [46 (92%) rural; 22 (77%) non-rural], cleaning employees [41 (82%) rural; 23 (82%) non-rural], receptionists [40 (80%) rural; 14 (50%) non-rural], and nurses [45 (90%) rural; 26 (93%) non-rural] in their staff (Table 1). At least half of the PHC centers included a health promotor, and less than half included a social worker (Table 1). Less than half of the PHC centers included GP trainees [15 (30%) rural; 6 (21%) non-rural] (Table 1), reporting one GP trainee each. Both in rural and non-rural areas, few PHC centers included a dietician, a psychologist, or a physiotherapist (Table 1).

**Table 1:** Characteristics of participating public PHC centers

PHC center characteristic	Rural PHC centers *N* = 50	Non-rural PHC centers *N* = 28	*P*-value^†^
Employees, median (IQR); (*N* = 75; 49 rural, 26 non-rural)	25 (6, 40)	23 (3, 61)	0.87
GPs, median (IQR); (*N* = 74; 50 rural, 24 non-rural)	6 (3, 9)	6 (1, 16)	0.93
PHC centers with practice manager, n (%)	40 (80%)	18 (64%)	0.18
PHC centers with dietician, n (%)	4 (8%)	1 (4%)	0.65
PHC centers with health promotor, n (%)	25 (50%)	16 (57%)	0.64
PHC centers with GP, n (%)	46 (92%)	22 (77%)	0.16
PHC centers with GP trainee, n (%)	15 (30%)	6 (21%)	0.44
PHC centers with physiotherapist /manual therapist /osteopath, n (%)	10 (20%)	5 (18%)	0.99
PHC centers with social worker, n (%)	11 (22%)	11 (39%)	0.12
PHC centers with cleaning employee, n (%)	41 (82%)	23 (82%)	0.99
PHC centers with receptionist, n (%)	40 (80%)	14 (50%)	0.01
PHC centers with psychologist, n (%)	3 (6%)	4 (14%)	0.24
PHC centers with nurse /nurse assistant, n (%)	45 (90%)	26 (93%)	0.99
PHC centers with limitations related to the building or the infrastructure that compromise high-quality and safe care since the pandemic, n (%)	18 (36%)	8 (29%)	0.50
PHC centers considering adjustments to the building or the infrastructure in the future due to the pandemic, n (%); (N = 75; 50 rural, 25 non-rural)	16 (32%)	7 (28%)	0.72
Size of PHC center population*, median (IQR); (*N* = 72; 49 rural, 23 non-rural)	15,000 (6250, 30,000)	33,000 (10,000, 50,000)	0.07

*PHC* primary health care, *IQR* interquartile range, *GP* general practitioner

†A P-value < 0.05 was considered as statistically significant

*Size was based on the number of registered patients; if that was not available, size was based on the total PHC center population

**Table 2:** PHC centers providing care for a population with the following characteristics above the country average

Population characteristic	Rural PHC centers *N* = 50	Non-rural PHC centers *N* = 28	*P*-value^†^
Migration background, n (%); (*N* = 71; 47 rural, 24 non-rural)	6 (13%)	5 (21%)	0.49
Limited /low health literacy, n (%); (N = 74; 49 rural, 25 non-rural)	6 (12%)	1 (4%)	0.41
Financial problems, n (%); (*N* = 73; 49 rural, 24 non-rural)	9 (18%)	4 (17%)	0.99
Psychiatric illness, n (%); (*N* = 69; 49 rural, 20 non-rural)	3 (6%)	0	0.55
Age older than 70 years, n (%); (N = 73; 49 rural, 24 non-rural)	24 (49%)	8 (33%)	0.22
Chronic conditions, n (%); (N = 74; 50 rural, 24 non-rural)	25 (50%)	6 (25%)	0.05
Limited social support /informal care, n (%); (*N* = 70; 50 rural, 20 non-rural)	7 (14%)	2 (10%)	0.99

*PHC* primary health care

†A P-value < 0.05 was considered as statistically significant

Due to the pandemic, about one third of PHC centers reported major limitations related to the building or infrastructure [18 (36%) rural; 8 (29%) non-rural] or considered future adjustments [16 (32%) rural; 7 (28%) non-rural]. PHC centers reported that they served, on average, a population of 15,000 (IQR 6250, 30,000) patients in rural, and 33,000 (10,000, 50,000) patients in non-rural areas.

Both rural and non-rural PHC centers less frequently reported that they served above the average vulnerable populations. Specifically, 6 (13%) PHC centers in rural and 5 (21%) in non-rural areas reported that they served a population including above the average patients with migration backgrounds (Table 2). Less than one-fifth of the PHC centers both in rural and non-rural areas reported that they served a population including above the average patients with limited or low health literacy, financial problems, psychiatric vulnerability, limited social support, or informal care (Table 2). However, a higher proportion of both rural and non-rural PHC centers reported they served a population including above the average elderly people, and patients with chronic conditions. Specifically, 24 (49%) PHC centers in rural and 8 (33%) in non-rural areas reported that they served a population including above the average patients over 70 years old (Table 2). Twenty-five (50%) PHC centers in rural and 6 (25%) in non-rural areas reported that they served a population including above the average patients with chronic conditions (Table 2).

### Organization of PHC centers during the pandemic

The number of PHC centers reporting that they implemented specific measures during the pandemic varied both in rural and non-rural settings in Greece. We considered measures reported by at least 75% of the PHC centers as “followed by the majority of the PHC centers” (Additional file 1 Table S1); those reported by 25–74% of the PHC centers as “followed by some of the PHC centers” (Additional file 1 Table S2); and those reported by less than 25% of either rural or non-rural PHC centers as “followed by few of the PHC centers” (Table 3). We focused more on measures followed by less than 25% of either rural or non-rural PHC centers (Table 3) to recognize potential areas of improvement.

**Table 3:** Measures followed by few of the PHC centers (reported by less than 25% PHC centers either in rural or in non-rural settings)

Reported measures	Rural PHC centers *N* = 50	Non-rural PHC centers *N* = 28	*P*-value^†^
*Patient flow*			
PHC centers with informing message in online appointment for complaints that can be solved without a visit to the PHC center, n (%) (*N* = 27; 14 rural, 13 non-rural)	3 (21%)	4 (31%)	0.68
PHC centers with video consultations use			
Before pandemic, n (%)	2 (4%)	0	0.53
Since pandemic, n (%)	6 (12%)	2 (7%)	0.70
PHC centers reporting, they compiled lists from EMR for patients with a chronic disorder, n (%) (*N* = 62; 42 rural, 20 non-rural)	2 (5%)	2 (10%)	0.59
PHC centers reporting that they check more than before whether patients (in)directly experienced domestic violence since pandemic, n (%) (N = 50; 36 rural, 14 non-rural)	3 (8%)	1 (7%)	0.99
PHC centers reporting that they check more than before whether patients experience financial problems since pandemic, n (%) (*N* = 49; 35 rural, 14 non-rural)	9 (26%)	1 (7%)	0.24
*Infection prevention*			
PHC centers with a tap operated with the elbow or with a movement detector, n (%)	10 (20%)	5 (18%)	0.99
PHC centers reporting that administrative documents were sent by postal mail for patients with (a suspicion of) COVID-19, n (%) (*N* = 61; 42 rural, 19 non-rural)	5 (12%)	4 (21%)	0.44
PHC centers reporting that administrative documents were sent by regular email for patients with (a suspicion of) COVID-19, n (%) (*N* = 67; 45 rural, 22 non-rural)	5 (11%)	8 (36%)	0.02
PHC centers reporting that administrative documents were sent through a secured server with a code for patients with (a suspicion of) COVID-19, n (%) (*N* = 57; 40 rural, 17 non-rural)	3 (8%)	3 (18%)	0.35
*Communication to patients*			
PHC centers reporting that they provided information in multiple languages in their websites (*N* = 43; 26 rural, 17 non-rural)	3 (12%)	0	0.27
PHC centers reporting that they had an answering machine that provided information in multiple languages (N = 50; 35 rural, 15 non-rural)	2 (6%)	0	0.99
PHC centers reporting that they had available leaflets that provided information in multiple languages (*N* = 55; 35 rural, 20 non-rural)	1 (3%)	0	0.99
PHC centers reporting that they had available leaflets that provided information on COVID-19 in multiple languages (N = 73; 49 rural, 24 non-rural)	3 (6%)	2 (8%)	0.65

*PHC* primary health care, *EMR* electronic medical records

^†^A P-value < 0.05 was considered as statistically significant

### Measures that the majority of PHC centers followed during the pandemic

Both rural and non-rural PHC centers reported several adjustments in the appointment system, triage, and referrals to support patient flow with safety during the pandemic. They allowed sufficient time between consultations for disinfecting the consultation room (Additional file 1 Table S1). They adopted a phone protocol for patients who called to make an appointment (Additional file 1 Table S1). They implemented triage protocols, and they engaged non-GP staff in giving information, and recommendations by phone, in explaining caregiver’s instructions to illiterate patients, and patients with limited health literacy, or migrants, and in the triage process (Additional file 1 Table S1). In case of referral, they invariably checked whether the patient had access to the specific health service (Additional file 1 Table S1). Infection prevention measures, including basic hygiene and disinfection of infrastructure, were generally in place in most PHC centers both in rural and non-rural areas (Additional file 1 Table S1).

### Measures followed by some PHC centers during the pandemic

The staff in rural PHC centers was more likely to arrange home visits in a way that COVID-19 patients were seen at the end of the round as compared to the staff in non-rural settings [29 (78%) in rural vs. 3 (27%) in non-rural; *P*-value 0.003] (Additional file 1 table S2). Rural PHC staff was also more likely to report checking whether a patient could isolate at home when necessary [37 (76%) in rural vs. 12 (48%) in non-rural; *P*-value 0.02] (Additional file 1 Table S2). Contacting home care services to inform patients for the diagnosis of a major infectious disease other than COVID-19 was more often reported in rural [31 (65%)] as compared to non-rural [7 (32%)] PHC centers (P-value 0.02) (Additional file 1 Table S2). On the contrary, in fewer rural PHC centers [27 (64%)], the staff reported transferring patient files to another colleague in case of GP sick leave, as compared to non-rural settings [21 (91%)] (P-value 0.02). Other measures reported by some PHC centers did not significantly differ between rural and non-rural settings (Additional file 1 Table S2). Specifically, the majority of both rural and non-rural PHC centers provided available walk-in hours for patients without an appointment [40 (80%) rural and 20 (74%) non-rural]. Twelve (67%) rural and 6 (36%) non-rural PHC centers reported that patients had to state a reason when making an online appointment. Twenty-four (51%) rural and 9 (38%) non-rural PHC centers reported contacting psychologically vulnerable patients. Twelve (27%) rural and 6 (26%) non-rural PHC centers reported that they contacted patients with previous problems of domestic violence or with a problematic child-rearing situation. (Additional file 1 Table S2).

Daily planned meetings to discuss directives were reported by 13 (26%) rural and 11 (39%) non-rural PHC centers. Support from other PHC centers when staff members had a sick leave was reported by 26 (53%) rural and 9 (36%) non-rural PHC centers. Promoting cooperation with other PHC centers during the pandemic was reported by 25 (52%) rural and 12 (46%) non-rural PHC centers (Additional file 1 table S2).

### Measures followed by few PHC centers during the pandemic

Certain measures were reported by less than one fourth of the PHC centers either in rural or in non-rural areas, generally without significant differences between rural and non-rural settings (Table 3). However, rural PHC centers were less likely to report that administrative documents were sent by regular email for patients with (a suspicion of) COVID-19 as compared to non-rural PHC centers [5 (11%) rural vs. 8 (36%) non-rural; *P*-value 0.02] (Table 3). A limited number of PHC centers [3 (21%) rural; 4 (31%) non-rural] provided an informing message in their online appointment system for complaints that could be solved without a visit in the PHC center. Very few PHC centers reported that they implemented video consultations either before [2 (4%) rural] or since the pandemic [6 (12%) rural; 2 (7%) non-rural] (Table 3). A limited number of PHC centers reported taking certain initiatives, including compiling lists from electronic medical records (EMR) for patients with a chronic illness [2 (5%) rural; 2 (10%) non-rural]; initiating a discussion with their patients about domestic violence and checking directly or indirectly whether their patients experienced domestic violence since the pandemic started [3 (8%) rural; 1 (7%) non-rural] (Table 3). Few PHC centers [9 (26%) rural; 1 (7%) non-rural] reported that they asked their patients whether they experienced financial problems since the start of the pandemic (Table 3).

Few PHC centers reported that they adopted a mode to handle administrative documents other than the typical in-person pick up, i.e., postal mail [5 (12%) rural; 4 (21%) non-rural], and e-mail through a secured server with a code [3 (8%) rural; 3 (18%) non-rural] (Table 3).

Finally, there were only 3 (12%) rural PHC centers that reported providing information on their website in multiple languages, and 2 (6%) rural PHC centers with an answering machine that provided information in multiple languages. One (3%) rural PHC center reported available leaflets in multiple languages for the patients, whereas 3 (6%) rural and 2 (8%) non-rural PHC centers reported available leaflets on COVID-19 in multiple languages (Table 3).

## Discussion

Our survey provided nationwide empirical data on the structure and processes 1 year after the pandemic had started from a random sample of public PHC centers in Greece. Rural and non-rural PHC centers reported that they generally included general practitioners (GPs), cleaning employees, receptionists, and nurses in their staff while other health professionals were scarcely reported. One-third of PHC centers reported major limitations related to the building or considering future adjustments; for example, the availability of a separate entrance for COVID-19 patients, or the construction of a separate examination room for COVID-19 patients. Almost half of the PHC centers in rural areas reported that they served a population including above-the-average patients over 70 years old and patients with chronic conditions. Several measures on patient flow, infection prevention, information processing, communication to patients, collaboration, and collegiality were implemented by PHC centers both in rural and non-rural areas. Noteworthy, rural PHC centers were more likely to report home visits for COVID-19 patients at the end of the round, to check whether a patient could isolate at home when necessary, and to contact home care services to inform patients for the diagnosis of infectious diseases other than COVID-19. On the contrary rural PHC centers were less likely than non-rural PHC centers to transfer patients’ files to colleagues when a GP had a sick leave, and to use regular e-mail to send administration documents to patients that were probably infected. Certain measures were reported by few PHC centers suggesting potential priority areas of improvement. Specifically, the use of online messages about complaints that can be solved without a visit to the PHC center, the use of video consultations with patients, and the use of electronic medical records (EMRs) to systematically identify the list of patients with chronic conditions were scarcely reported. In addition, very few PHC centers reported measures to support identifying and reaching out to vulnerable population, including patients that may have experienced domestic violence, financial problems, mental health issues, or a problematic child-rearing situation. Finally, providing administrative documents to patients through postal, or electronic mail as well as information in multiple languages through the PHC website, an answering machine, or leaflets were lacking in most PHC centers both in rural and non-rural settings.

In our survey, we captured information on issues related to the dimensions of quality of care, such as safety, patient-centeredness, accessibility, and equity [18, 19]. To ensure patient safety, PHC centers in Greece largely adopted a variety of measures, which included infection prevention measures, appointment system adaptations, triage based on suggested protocols, reorganization of consultations, and infrastructure adjustments. Similar adaptations in the PHC service delivery process were documented worldwide [20–22] and have also been described in previous publications in Greece [23]. These efforts focused on successfully preventing virus transmission. However, safety may have been challenged for non-COVID-19 patients. For example, patients with other urgent conditions may have preferred to follow the general “stay at home” recommendation and postponed care. Noteworthy, emergency department visits during the first 9 months of the COVID-19 pandemic reduced more than 33% [24]. Similarly, previous reports suggested low attendance rates in PHC centers in Greece and an excess of non-COVID-19 mortality during the first months of the pandemic [24]. In addition, phone communication alone may not be helpful for certain patients. Thus, well-established protocols to facilitate the assessment of patients through video calls and adequately trained health care teams to provide home care services might have increased the delivery of PHC services for patients who chose not to visit PHC centers [25, 26].

Our survey showed that most PHC centers in Greece based their services on a team including GPs, nurses, health managers, and cleaning staff. This traditional model may be important to care for patients with acute illnesses or specific chronic health issues. However, it may not be sufficient to provide integrated care. The low proportion of PHC centers that included other health care professionals, e.g., social workers, phycologists, physiotherapists, etc., might have precluded a large part of PHC in Greece from forming multidisciplinary teams and achieving adequate integration with social services. Based on our survey, we should acknowledge that the pandemic outbreak forced non-GP staff to undertake additional tasks, representing a first step toward team-based care [27]. The latter was of great importance, considering that nursing staff in PHC centers in Greece invariably operated within a restricted task-oriented framework [28, 29]. With attendance rates dropping significantly, several PHC centers reported actively reached out to patients who postponed healthcare or contacting patients with known chronic conditions who needed follow-up care. However, very few PHC centers reported that they systematically compiled lists from EMRs for patients with a chronic disorder. A likely explanation for that might be that, despite several efforts [30], the implementation of EMRs in the Greek healthcare setting had been widely fragmented until the COVID-19 pandemic [14]. EMRs can be used to pre-screen patient needs, and identify high-risk patients and those with gaps in care while offering the possibility for multidisciplinary teams to coordinate care and co-manage patients with complex needs [31, 32]. Replacing traditional paper-based medical records by user-friendly EMRs may prove a valuable population health management tool for PHC centers in Greece. Current efforts to address all potential barriers should be encouraged and rapidly implemented [14].

Our survey reported the PHC center population size did not significantly differ between rural and non-rural areas. One might expect that rural settings might have a registered population of smaller size as compared to non-rural settings. However, our findings did not support that, which is likely due to the absence of a registered population in most PHC centers and the lack of a well-defined catchment area [33]. Without a registered population in most PHC centers, potential gaps in population coverage were likely to be under-reported. Moreover, before the pandemic, PHC centers in Greece did not use interoperable systems [8], and the possibility of video calls was limited [13]. Health professionals in rural settings have suggested the introduction of e-health to provide high-quality healthcare to their patients [29]. Promoting paperless, remote, electronic prescriptions to patients was a promising step in that direction [14]. However, since the pandemic outbreak, unlike in other countries [20–22], video consultations were rarely implemented in Greece as an alternative for delivering remote care to COVID-19 and non-COVID-19 patients when appropriate [34]. The absence of prior experience conducting video consultations may have played a role in this observation. In addition to adequate training and infrastructure, well-established guidelines for remotely delivering health care services and a specific compensation framework for health professionals might have contributed to wider use of virtual care, minimizing health inequalities for certain patient groups [35].

Health equity is a notion closely associated with social determinants of health, which may hinder care for all as a source of health disparities. Health care providers should screen and identify social determinants of health for their patients, preferably in the context of collaborative models of care [31]. Besides a health crisis, the COVID-19 pandemic has also been a socio-economic crisis, disproportionally affecting vulnerable groups [36, 37]. The need to address the mental and social needs of the population directly or indirectly affected by COVID-19 has been highly emphasized [38]. Supporting current successful approaches of care, i.e., care by mobile mental health units, by developing collaborative models with PHC professionals may help address the unmet needs of patients with mental illness [39]. In the PHC setting, addressing social determinants of health is a prerequisite to achieving improved health outcomes [40]. In our survey, most PHC centers did not report checking for vulnerable patients during the pandemic. This observation could imply that these patient groups might have experienced difficulties in accessing PHC centers [41]. Another explanation might be that PHC providers lack sufficient motivation, or training, to implement this action in everyday practice [42]. Considering that certain events, i.e., domestic violence incidents, increased during the pandemic [43], the need for further training of PHC professionals in screening and providing collaborative care for vulnerable patients should be included among essential clinical skills [44]. In our survey, identifying patients with multi-morbidity as well as elderly patients was reported more frequently. This was likely due to the disease-oriented approach adopted in several PHC settings. Noteworthy, previous work reported that patients with chronic diseases in Greece had described a better experience with PHC services than patients without a chronic condition [45].

Earlier reports had brought up the issue of fragmented PHC and its consequences on the continuity of the delivery of care [46]. The demand for an urgent response to the pandemic brought several of these consequences to the surface despite the enormous efforts both by PHC employees and policymakers [47]. Our findings may highlight issues with high priority for PHC centers in Greece. As one-third of PHC centers reported major limitations related to the building or considering future adjustments, maintaining adequate infrastructure in PHC centers is a prerequisite. In addition, supporting human resources by establishing a quality management approach to ensure the identification of barriers and work on continuous improvement with measurable outcomes may also be considered [18]. To facilitate the work in PHC centers, the update of continuing health professional educational programs, when necessary, should be encouraged. PHC professionals should be motivated to get familiar with EMR use and its potential for assessing the needs of the registered population. They should also be adequately prepared to organize and implement modern models of care based on the needs of their patients. Specialized care delivered either in hospitals or by non-generalists in the community as well as by other health professionals may be integrated into collaborative care models run by PHC teams [48, 49]. A step toward that direction has already been made by integrating public health services with PHC teams to ensure vaccination for COVID-19 among patients with limited access to health services [49].

Our study had several limitations. First, it was an online survey, and therefore, we based our findings on what was reported without being able to verify them. Moreover, since there is no official list that identifies rural, and non-rural PHC centers in Greece, it would be difficult to check on the representativeness of our sample based on the rurality criterion. However, we collected a high proportion of nationwide responses in our random sample, which might support the generalizability of our results for PHC centers in Greece. Second, our results on the comparison between rural and non-rural PHC centers were based on secondary analyses; and thus, potential significant differences should be cautiously interpreted. In addition, we excluded non-government-funded PHC settings since no updated list was available to use for selecting a random sample. Thus, whether structures and processes might have been different during the pandemic in the private PHC sector remains unclear. Finally, our work was based on survey data; thus, we cannot elicit causal inferences from our results.

## Conclusions

Our survey provided a snapshot of the situation among PHC centers in Greece and their organization 1 year after the COVID-19 pandemic breakout. Overall, the pandemic spotlighted the longstanding deficiencies of Greece’s PHC. However, it would be misleading not to mention that it has also forced PHC centers to mobilize internal forces and develop new measures. Future changes in Greece’s health policy should enable a health care delivery setting that offers the prerequisites for implementing the Astana Declaration principles, considering PHC as an inspirational vision and set of values for health development [50].

### Supplementary Information

**Supplementary Material 1.**

**Supplementary Material 2.**

## Data Availability

The anonymized data is held at Ghent University and is available to participating partners for further analysis upon signing an appropriate data transfer agreement.

## Abbreviations

COVID-19: Coronavirus Disease 2019
EFPC: European Forum for Primary Care
EGPRN: European General Practice Research Network
EMR(s): Electronic Medical Record(s)
EQuiP: European Society for Quality and Patient Safety in General Practice/Family Medicine
GP(s): General Practitioner(s)
IQR: Interquartile Range
PHC: Primary Health Care
REDCap: Research Electronic Data Capture
WONCA: World Organization of Family Doctors
