# Supplementary material for: Measures during the COVID-19 pandemic in public primary health care in Greece: is there still a missing link to universal health coverage?
Source: BMC Prim Care. 2024 May 17;24(Suppl 1):287. doi: 10.1186/s12875-024-02392-7 (PMC11100090; doi:10.1186/s12875-024-02392-7)
Supplement: Supplementary file 1 — Supplementary Material 1. [file 12875_2024_2392_MOESM1_ESM.docx]

**Table S1.** Measures followed by the majority of the PHC centers (reported by at least 75% of the PHC centers both in rural and in non-rural settings)

| **Reported measures** | **Rural PHC centers**  **N= 50** | **Non-rural PHC centers**  **N= 28** | **P-value**^†^ |
| --- | --- | --- | --- |
| *Patient flow* |  |  |  |
| PHC centers with sufficient time between consultations for disinfection of the consultation room, n (%) (N=77; 49 rural, 28 non-rural) | 41 (84%) | 23 (82%) | 0.99 |
| PHC centers with available protocol for answering phone calls from potential COVID-19 patients, n (%) (N=77; 50 rural, 27 non-rural)* | 44 (88%) | 26 (96%) | 0.41 |
| PHC centers that use the available phone protocol n (%) (N=69; 44 rural, 25 non-rural) | 43 (98%) | 24 (96%) | 0.99 |
| PHC centers with GP support, if telephonic triage is performed by someone other than a GP, n (%) (N=73; 48 rural, 25 non-rural) | 44 (92%) | 21 (84%) | 0.43 |
| PHC centers with recent triage information available in every consultation room, n (%) (N=68; 45 rural, 23 non-rural)** | 42 (93%) | 18 (78%) | 0.11 |
| PHC centers where non-GP staff is more involved in giving information and recommendations by phone, n (%) (N=73; 47 rural, 26 non-rural) | 38 (81%) | 25 (96%) | 0.09 |
| PHC centers where non-GP staff is more involved in giving information to or explaining what a caregiver has said to illiterate patients, patients with low health literacy or migrants, n (%) (N=75; 48 rural, 27 non-rural) | 36 (75%) | 25 (93%) | 0.07 |
| PHC centers where non-GP staff is more involved in the triage, n (%) (N=75; 47 rural, 28 non-rural) | 41 (82%) | 23 (87%) | 0.74 |
| PHC centers check whether a patient can access another facility when he/she is referred, n (%) | 48 (96%) | 26 (93%) | 0.62 |
| *Infection prevention* |  |  |  |
| PHC centers with a sink available, n (%) | 48 (96%) | 26 (92%) | 0.62 |
| PHC centers with a trash opened without contact, n (%) | 49 (98%) | 26 (93%) | 0.29 |
| PHC centers with disposable gloves, n (%) | 50 (100%) | 28 (100%) | NA |
| PHC centers with disposable coats, n (%) | 41 (82%) | 24 (86%) | 0.76 |
| PHC centers with surface disinfectant, n (%) | 48 (96%) | 27 (96%) | 0.54 |
| PHC centers with a paper to cover the examination table, n (%) (N=77; 49 rural, 28 non-rural) | 47 (96%) | 27 (96%) | 0.99 |
| PHC centers with a detailed cleaning protocol |  |  |  |
| Before pandemic, n (%) (N=76; 48 rural, 28 non-rural) | 27 (56%) | 15 (54%) | 0.99 |
| Since pandemic, (%) (N=75; 48 rural; 27 non-rural) | 41 (85%) | 24 (89%) | 0.99 |
| PHC centers with a hand sanitizer placed in consultation room |  |  |  |
| Before pandemic, n (%) | 33 (66%) | 18 (64%) | 0.99 |
| Since pandemic, n (%) | 49 (98%) | 26 (93%) | 0.29 |
| PHC centers using hand sanitizer during home visits |  |  |  |
| Before pandemic, (%) (N=57; 42 rural, 15 non-rural) | 19 (45%) | 7 (47%) | 0.99 |
| Since pandemic, (%) (N=55; 42 rural, 13 non-rural) | 36 (86%) | 10 (77%) | 0.43 |
| PHC centers with a hand sanitizer placed at the door or waiting room of the PHC center |  |  |  |
| Before pandemic, (%) (N=75; 48 rural, 27 non-rural) | 25 (52%) | 11 (41%) | 0.47 |
| Since pandemic, n (%) (N=77; 50 rural, 27 non-rural) | 48 (96%) | 25 (93%) | 0.61 |

PHC, primary health care; GP, general practitioner

^†^A P-value <0.05 was considered as statistically significant

*Protocols included were based on government guidelines (N=64; 91%), and non-government instructions (N=6; 9%)

**Triage information might have been in print (N=55; 92%) or electronically (N=5; 8%)

**Table S2.** Measures followed by some of the PHC centers (reported by 25% to 74% both in rural and in non-rural settings)

| **Reported measures** | **Rural PHC centers**  **N= 50** | **Non-rural PHC centers**  **N= 28** | **P-value**^†^ |
| --- | --- | --- | --- |
| *Patient flow* |  |  |  |
| PHC centers with available walk-in hours for patients without an appointment, n (%) (N=77; 50 rural, 27 non-rural) | 40 (80%) | 20 (74%) | 0.58 |
| PHC centers where patients must state a reason when making appointment by phone, n (%) (N=65; 42 rural, 23 non-rural) | 36 (86%) | 15 (65%) | 0.07 |
| Patients with an appointment are called beforehand if it is unclear whether they pose a risk for infection, n (%) (N=66; 40 rural, 26 non-rural) | 28 (70%) | 16 (62%) | 0.60 |
| PHC centers where non-GP staff is more involved in actively reaching out patients who might postpone healthcare, n (%) (N=73; 46 rural, 27 non-rural) | 28 (61%) | 19 (70%) | 0.46 |
| PHC centers where GPs are more involved in actively reaching out patients who postpone healthcare, n (%) (N=68; 43 rural, 25 non-rural) | 29 (67%) | 19 (76%) | 0.58 |
| PHC centers contacted patients with chronic conditions who needed follow up care, n (%) (N=74; 47 rural, 27 non-rural) | 32 (68%) | 16 (59%) | 0.46 |
| PHC centers where patients must state a reason when making online appointment, n (%) (N=35; 18 rural, 17 non-rural) | 12 (67%) | 6 (36%) | 0.09 |
| PHC centers where COVID-19 patients are seen at the end of the round in home visits, n (%) (N=48; 37 rural, 11 non-rural) | 29 (78%) | 3 (27%) | 0.003 |
| PHC centers they check whether a patient can isolate at home, when necessary, n (%) (N=74; 49 rural, 25 non-rural) | 37 (76%) | 12 (48%) | 0.02 |
| PHC centers contacted psychologically vulnerable patients, n (%) (N=71; 47 rural, 24 non-rural) | 24 (51%) | 9 (38%) | 0.32 |
| PHC centers contacted patients with previous problems of domestic violence or with a problematic child-rearing situation, n (%) (N=67; 44 rural, 23 non-rural) | 12 (27%) | 6 (26%) | 0.99 |
| *Infection prevention* |  |  |  |
| PHC centers using a separate medical bag for home visits to patients with suspected infection |  |  |  |
| Before pandemic, (%) (N=55; 41 rural, 14 non-rural) | 23 (56%) | 6 (43%) | 0.54 |
| Since pandemic, n (%) (N=54; 41 rural, 13 non-rural) | 32 (78%) | 8 (62%) | 0.29 |
| Contact of home care services to inform patients for COVID-19 diagnosis, n (%) (N=71; 49 rural, 22 non-rural) | 37 (76%) | 12 (55%) | 0.10 |
| Contacting home care services to inform patients for the diagnosis of a major infectious disease other than COVID-19, n (%) (N=70; 48 rural, 22 non-rural) | 31 (65%) | 7 (32%) | 0.02 |
| *Information processing* |  |  |  |
| Protected time for reviewing guidelines and literature |  |  |  |
| Before pandemic, n (%) (N=77; 50 rural, 27 non-rural) | 23 (46%) | 15 (54%) | 0.20 |
| Since pandemic, n (%) (N=77; 50 rural, 27 non-rural) | 26 (52%) | 18 (64%) | 0.11 |
| PHC centers reporting daily planned meetings to discuss existing, new, or amended directives |  |  |  |
| Before pandemic, n (%) | 5 (10%) | 2 (7%) | 0.99 |
| Since the pandemic (N=76; 48 rural, 28 non-rural) | 13 (26%) | 11 (39%) | 0.44 |
| *Collaboration and collegiality* |  |  |  |
| PHC centers transferring patient files to another colleague in case of GP sick leave, n (%) (N=65; 42 rural, 23 non-rural) | 27 (64%) | 21 (91%) | 0.02 |
| Distributing the work so that the well-being of colleagues was not compromised when staff members had a sick leave due to COVID-19, n (%) | 30 (60%) | 19 (68%) | 0.63 |
| PHC centers reporting support from other PHC centers when staff members had a sick leave, n (%) (N=74; 49 rural, 25 non-rural) | 26 (53%) | 9 (36%) | 0.22 |
| PHC centers reporting that potential incidents of quality of care are discussed at team meetings on a regular basis, n (%) (N=75; 48 rural; 27 non-rural) | 24 (50%) | 9 (33%) | 0.16 |
| PHC centers reporting that COVID-19 promoted cooperation with other PHC centers, n (%) (N=74; 48 rural, 26 non-rural) | 25 (52%) | 12 (46%) | 0.81 |

PHC, primary health care; GP, general practitioner

^†^A P-value <0.05 was considered as statistically significant
